# Solitary fibrous tumor of the sublingual gland: a case report

**DOI:** 10.3389/froh.2026.1742254

**Published:** 2026-02-03

**Authors:** Jinpeng Liu, Yao Yu, Dongliang Lin, Yaoxiang Xu, Lei Ma

**Affiliations:** 1 The Affiliated Hospital of Qingdao University, Qingdao, China; 2 School of Stomatology, Qingdao University, Qingdao, China; 3 Dental Digital Medicine & 3D Printing Engineering Laboratory of Qingdao, Qingdao, China

**Keywords:** case report, floor of mouth mass, immunohistochemistry, solitary fibrous tumor, sublingual gland tumor

## Abstract

**Objectives:**

To emphasize the importance of considering solitary fibrous tumor (SFT) in the differential diagnosis of sublingual gland masses, particularly those presenting with unusual symptoms like tongue deviation.

**Methods:**

A case of a 41-year-old female with a painless sublingual mass and tongue deviation was analyzed. Clinical presentation, imaging, surgical management, histopathological findings, immunohistochemical results, and follow-up were systematically reviewed. Management consisted of complete surgical excision of the sublingual gland and the tumor, with preservation of the lingual and hypoglossal nerves.

**Results:**

Histopathology revealed haphazardly arranged spindle cells with characteristic staghorn-shaped blood vessels. Immunohistochemical analysis confirmed strong nuclear STAT6 and diffuse CD34 positivity, consistent with SFT. The patient remained recurrence-free at 18 months postoperatively, although tongue deviation persisted.

**Conclusions:**

sublingual gland SFTs are exceptionally rare and may mimic malignancies. Benign tumors such as SFT should be considered in the differential diagnosis of sublingual masses. Definitive diagnosis depends on histopathological and immunohistochemical confirmation. Complete surgical excision offers a favorable prognosis.

## 1 Introduction

Solitary fibrous tumor (SFT) is a rare mesenchymal tumor originating from fibroblasts, first described by Klemperer et al. in 1931 (1). The most common site for SFT is the pleura, with rare occurrences in the head and neck region (2). SFTs of the head and neck account for approximately 6% of all cases, with the nasal cavity, orbit, oral cavity, oral mucosa, and salivary glands being the most frequently affected sites (3). SFTs located in the sublingual gland are extremely rare, with only five reported cases to date (4–8). Moreover, tumors of the sublingual gland have a low incidence, with 90% being malignant, most commonly adenoid cystic carcinoma, which often invades nerves, leading to symptoms such as tongue numbness and deviation (9). This case reports a rare sublingual gland SFT, and the clinical presentation of tonguedeviation significantly complicated the diagnosis. The report provides valuable insights for the diagnosis and management of sublingual gland tumors.

## 2 Case description

**Figure 1:**
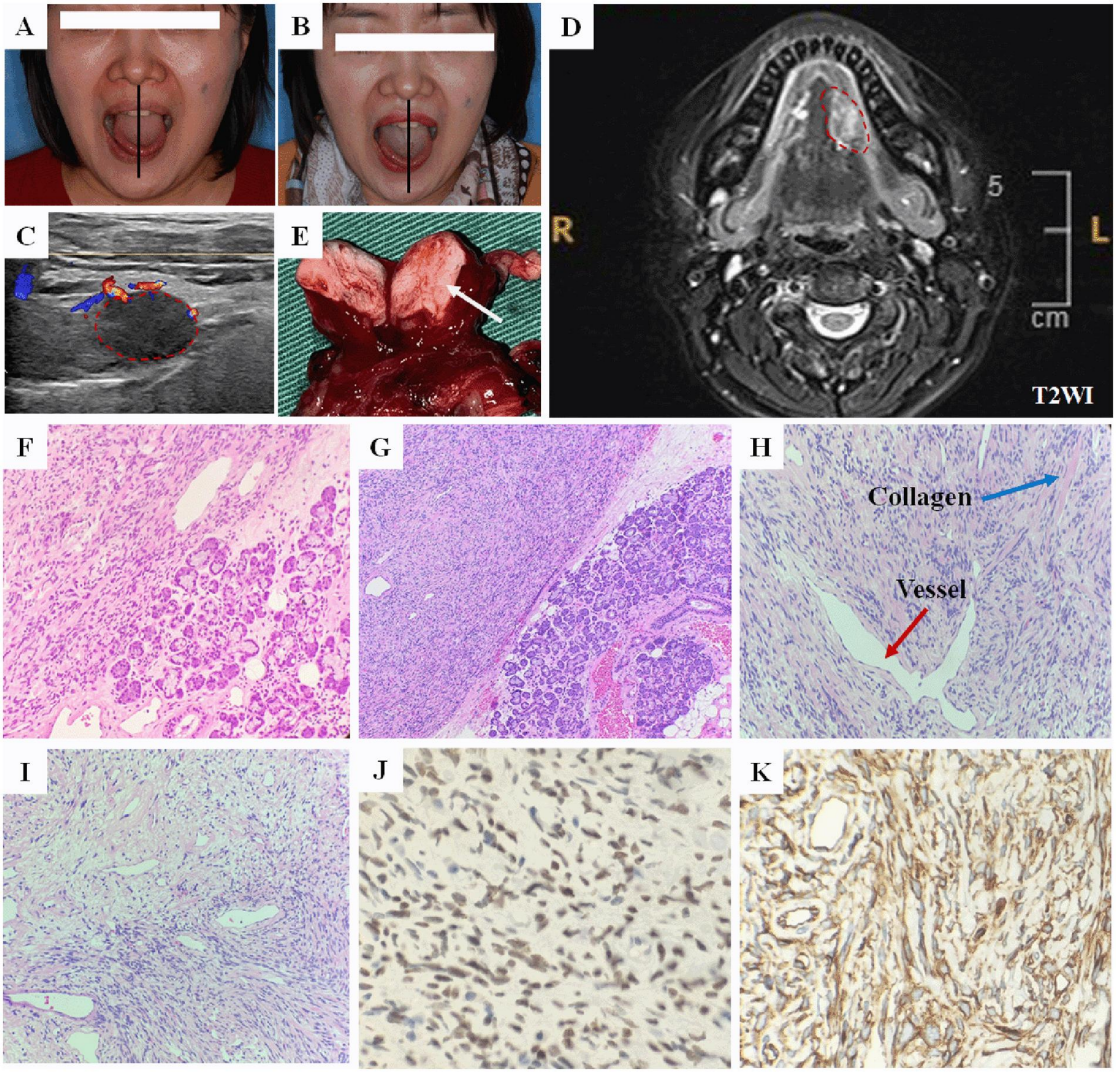
Case presentation. **(A)** Preoperative photo, tongue deviation to the left. **(B)** The tongue remained deviated to the left 18 months postoperatively. **(C)** Ultrasound revealed a 1.9 cm × 1.5 cm hypoechoic nodule in the left floor of the mouth. **(D)** MRI demonstrated a mixed high-signal lesion on T2-weighted images (T2WI). **(E)** The gross specimen appeared oval-shaped, grayish-white, and firm on sectioning. **(F)** HE staining (100×) showing the clear boundary between the tumor and normal sublingual gland. **(G)** HE staining (200×) highlighting the distinct interface and expansile growth pattern. **(H)** HE staining (200×) exhibiting characteristic staghorn-shaped blood vessels (red arrow) and keloid-type collagen deposition (blue arrow). **(I)** HE staining (200×) displaying alternating hypercellular and hypocellular areas composed of haphazardly arranged spindle cells in a collagenous stroma. **(J)** Immunohistochemistry (400×) for STAT-6 shows diffuse nuclear positivity. **(K)** Immunohistochemistry (400×) for CD34 shows diffuse positivity.

A 41-year-old female presented with a painless mass in the left floor of the mouth, which had been gradually enlarging for three years, and sought treatment at the Affiliated Hospital of Qingdao University. The patient had no significant medical history and denied any hereditary conditions. On examination, a 1.8 cm × 1.5 cm painless and firm mass was palpable in the left floor of the mouth, with good mobility. The tongue showed no numbness but exhibited left-sided deviation (Figure 1A), raising concern for potential hypoglossal nerve involvement or mechanical displacement by the tumor. No significant lymphadenopathy was observed in the neck. Ultrasound revealed a 1.9 cm × 1.5 cm hypoechoic nodule in the left floor of the mouth, with prominent blood flow signals at the margins (Figure 1C). MRI demonstrated a low-signal lesion on T1-weighted images (T1WI) and a mixed high-signal lesion on T2-weighted images (T2WI), with areas of longer T2 signal within. The lesion's maximum diameter was 15.6 mm, and there was displacement of the adjacent tongue muscles (Figure 1D). Based on the clinical and imaging findings, the differential diagnosis included salivary gland tumors (e.g., pleomorphic adenoma, adenoid cystic carcinoma), neurogenic tumors (e.g., schwannoma), and soft tissue neoplasms (e.g., leiomyoma). Malignancy such as adenoid cystic carcinoma was strongly considered due to the tongue deviation, which could mimic nerve invasion. Due to the proximity of the tumor to blood vessels, fine needle aspiration (FNA) biopsy was not performed. During surgery, the tumor appeared well-circumscribed, and there was no adhesion to the hypoglossal nerve, lingual nerve, or the sublingual duct. Both nerves were preserved, and the sublingual gland and tumor were completely excised. The gross specimen appeared oval-shaped, grayish-white, and firm on sectioning (Figure 1E). The intraoperative frozen section was inconclusive, but a benign nature was suspected. Histologically, HE staining at 100× magnification revealed a well-demarcated boundary between the tumor and the adjacent normal sublingual gland tissue (Figure 1F). At 200× magnification, the interface appeared distinct, confirming an expansile growth pattern without infiltration into the glandular parenchyma (Figure 1G). The tumor interior displayed alternating hypercellular and hypocellular areas composed of haphazardly arranged spindle cells in a collagenous stroma (Figure 1I), accompanied by characteristic staghorn-shaped blood vessels and keloid-type collagen deposition (Figure 1H). Immunohistochemical analysis showed strong nuclear STAT6 positivity (+), diffuse CD34 positivity (+), Ki-67 proliferation index ≤ 2%, and negative expression for S100 and SMA (Figures 1J,K). The final diagnosis was solitary fibrous tumor. Given the absence of necrosis and low mitotic activity (<4/10 HPF), the tumor was assessed to have a low risk for recurrence. The patient did not experience tongue numbness, bleeding, or infection postoperatively. At the 18-month follow-up, no local recurrence or metastasis was observed, although tongue deviation persisted (Figure 1B). The patient expressed significant relief after the successful, complete excision of the mass and was satisfied with the rapid recovery and the absence of tumor recurrence during the 18-month follow-up period, despite the persistence of the tongue deviation.

## 3 Discussion

SFTs have a low incidence, accounting for less than 2% of all soft tissue tumors (10). They are most commonly found in the pleura, representing approximately 30%–40% of all cases, and are also frequently observed in the meninges, abdominal cavity, and orbit. Primary occurrences in the oral cavity are less than 5%,10 with cases originating in the sublingual gland being extremely rare. Studies have shown that the development of SFT is closely associated with NAB2-STAT6 gene fusion, which leads to abnormal nuclear aggregation and expression of STAT6. STAT6 is a core diagnostic marker for SFT, with a positivity rate approaching 95%, exhibiting high sensitivity and specificity (11).

SFTs predominantly occur in middle-aged individuals between 40 and 60 years of age, with no significant gender differences (12). SFTs typically present as slow-growing, painless masses, which may cause local clinical symptoms when they compress adjacent tissues or invade nerves (13). In this case, the patient presented with tongue deviation, initially suggesting hypoglossal nerve involvement by the tumor. However, intraoperative findings revealed that the tumor was located in the superficial sublingual gland, with clear boundaries and distant from the hypoglossal nerve. Pathological examination confirmed a benign nature, thus excluding the possibility of nerve invasion. The differential diagnosis was made through medical history and cranial MRI, ruling out trauma, infection, and intracranial tumors. The patient's tongue deviation persisted 18 months postoperatively, and it was hypothesized that the symptom might be related to the patient's habitual behavior. We will continue to closely monitor this symptom. While preoperative diagnosis is typically crucial, in this case, the tumor's proximity to blood vessels made fine needle aspiration biopsy risky due to potential bleeding, and the tumor's malignant nature could not be excluded. Additionally, biopsy could lead to seeding or metastasis, thus a complete excision of the tumor was performed, with further resection considered based on intraoperative frozen section results.

SFTs exhibit atypical imaging features, making them difficult to diagnose through MRI, CT, or ultrasound. However, studies have shown that vascular proliferation is a significant characteristic of SFTs (14). Histologically, SFTs are characterized by haphazardly arranged spindle-shaped tumor cells, with evenly distributed nuclear chromatin. The stroma often contains characteristic staghorn-shaped blood vessel branches and collagen deposition (15). Immunohistochemically, the combination of diffuse CD34 positivity and specific nuclear STAT6 expression constitutes the essential diagnostic criteria for SFTs (11, 16). In this case, both markers were positive. Sublingual gland SFTs must be differentiated from schwannomas, pleomorphic adenomas, leiomyomas, and adenoid cystic carcinomas. SFTs typically show strong STAT6 positivity, whereas other tumors are usually negative.

SFTs are tumors with intermediate biological behavior. Although the majority are benign, approximately 10% are malignant (6). Malignant SFTs typically present with increased tumor cell density, marked atypia, a mitotic index ≥4 per 10 high-power fields (HPF), tumor necrosis, and infiltrative borders (13). Most patients with SFT have a favorable prognosis; however, 10%–20% of cases may recur or metastasize. SFTs with a mitotic index ≥4 per 10 HPF are associated with a higher risk of recurrence and metastasis (17).

The treatment of SFTs should be tailored based on tumor location, biological behavior, and clinical staging. For localized and resectable tumors, surgical excision within safe margins is the primary treatment approach (18). When surgical margins are positive or the tumor is located in high-risk areas, radiation therapy is often used as an adjunct (19). Chemotherapy is not effective for advanced metastatic SFTs, and angiogenesis inhibitors such as pazopanib are recommended as targeted therapies. Immunotherapy has shown promise in recent studies but remains in the exploratory phase, with its clinical efficacy yet to be determined (20).

## 4 Conclusion

In conclusion, sublingual gland SFTs are extremely rare. When a slow-growing, painless tumor is present in the sublingual gland, clinicians should consider SFT in the differential diagnosis. Additionally, sublingual gland tumors associated with tongue deviation are not necessarily malignant, and other potential causes such as poor habits, compression, or intracranial tumors should be excluded. There are limited data on the clinical presentation, imaging, and treatment of sublingual gland SFT, necessitating further research with more data and long-term follow-up.

## Data Availability

The raw data supporting the conclusions of this article will be made available by the authors, without undue reservation.
